# Supplementary material for: Antibiotic Resistance Rates for Helicobacter pylori in Rural Arizona: A Molecular-Based Study
Source: Microorganisms. 2023 Sep 12;11(9):2290. doi: 10.3390/microorganisms11092290 (PMC10536767; doi:10.3390/microorganisms11092290)

Analysis of clarithromycin sensitivity.

Using a Viasure kit, we analyzed all biopsy samples collected for the parent study, including those with a history of *H. pylori* infection. This method relied on identifying *H. pylori*-positive samples and their subsequent amplification of the 23S ribosomal gene after column purification of the PCR product and sequencing by a commercial company (Genewiz, Inc.).

The kit measures resistance to clarithromycin by targeting two known mutations, (A2142G and A2143G). The kit used four sets of primers:1) clarithromycin-resistance coupled to FAM dye (6-Carboxyfluorescein (blue); 2) wild-type coupled to HEX dye (green); 3) presence of *H. pylori* coupled to ROX dye (red); and 4) an internal control (actin) coupled to Cy5 dye (Cyanine-5-purple).

This kit identified 100% of the clarithromycin-positive samples with A2142G and A2143G mutations. The kit also identified 100% of clarithromycin-positive samples as A2142G/A2143G with another mutation, T2182C. When the mutation T2182C was analyzed alone, it was not recognized by the kit (Supplemental Table S1). Supplemental Figure S1 shows a PCR run in which amplification of the FAM dye corresponds to the reaction for clarithromycin resistance. The kit eliminates any potential false positives when the same sample is amplified by the HEX dye, indicating that this sample is not clarithromycin-resistant and behaves like a wild-type strain.

Table S1. Testing of clarithromycin-resistant *H. pylori* using the commercial kit from Viasure that measures resistance to clarithromycin by targeting two known mutations, A2142G and A2143G.

		**Viasure Real Time PCR Kit**	
**Mutations**	**N**	**Positive**	**Negative**
T2182C	**28**	0	100%
A2142G	**5**	100%	0%
A2143G	**4**	100%	0%
A2142G+T2182C	**1**	100%	0%
A2143G+T2182C	**4**	100%	0%
10 Hp (+) samples	**10**	100% (6/10)	(4/10)
		(Confirmed by sequencing)	
Hp (-)	40	0%	100%

Figure S1. PCR runs for clarithromycin-positive *H. pylori* detected in the FAM channel (Blue). Arrow indicates a suspicious sample. In addition, a wild-type phenotype was detected in the HEX channel, which confirmed the lack of clarithromycin resistance in this sample. Data was collected using the clarithromycin-resistant *H. pylori* commercial kit from ViaSure.

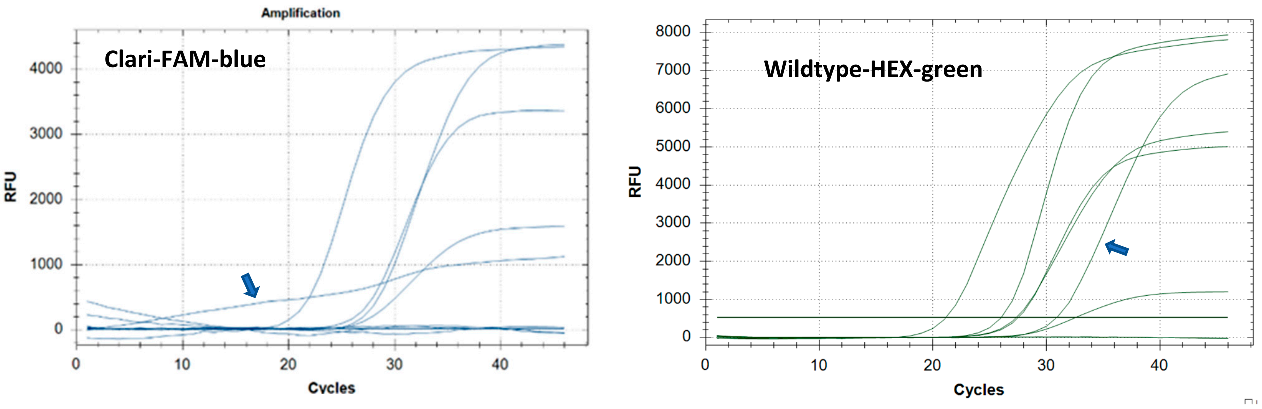

Supplement: Supplementary file 1 [file microorganisms-11-02290-s001.zip › microorganisms-2505951-supplementary.pdf]
